# The Flame-Retardant Mechanisms and Preparation of Polymer Composites and Their Potential Application in Construction Engineering

**DOI:** 10.3390/polym14010082

**Published:** 2021-12-27

**Authors:** Jingjing Shen, Jianwei Liang, Xinfeng Lin, Hongjian Lin, Jing Yu, Shifang Wang

**Affiliations:** 1 School of Civil Engineering and Architecture, Taizhou University, Taizhou 318000, Zhejiang, China; Linhj2011@tzc.edu.cn (H.L.); yujing79@tzc.edu.cn (J.Y.); mr_wsf@tzc.edu.cn (S.W.); 2 Building Office, Taizhou Urban and Rural Planning & Design and Research Institute Co., Ltd., Taizhou 318000, China; leiting_ye@tzc.edu.cn

**Keywords:** polymer composites, flammability, flame-retardant composites, construction materials

## Abstract

Against the background of people’s increasing awareness of personal safety and property safety, the flame retardancy (FR) of materials has increasingly become the focus of attention in the field of construction engineering. A variety of materials have been developed in research and production in this field. Polymers have many advantages, such as their light weight, low water absorption, high flexibility, good chemical corrosion resistance, high specific strength, high specific modulus and low thermal conductivity, and are often applied to the field of construction engineering. However, the FR of unmodified polymer is not ideal, and new methods to make it more flame retardant are needed to enhance the FR. This article primarily introduces the flame-retardant mechanism of fire retardancy. It summarizes the preparation of polymer flame-retardant materials by adding different flame-retardant agents, and the application and research progress related to polymer flame-retardant materials in construction engineering.

## 1. Introduction

In recent years, fire accidents have occurred frequently, causing serious losses to people’s lives and of property. Hence, it is necessary to develop high-efficiency flame-retardant materials. [1,2,3,4]. In general, unmodified polymer materials do not have ideal flame retardant properties. The limiting oxygen index (LOI) of common plastics such as polyethylene (PE) and polypropylene (PP) is only 17.0–18.0%. PP and PE are flammable or combustible materials [5]. Because the main chain of some engineering plastics contains nitrogen, sulfur, silicon, fluorine and other flame retardant elements, their LOI has been improved but still cannot meet the requirements of building flame retardancy. For example, the LOI of polyamide is 24–28%, and it has the ability to self-extinguish when on fire. However, it is still unable to effectively prevent the spread of fire when it is used as an insulating layer of cables or wires. Phenolic foam, polystyrene foam and polyurethane foam have excellent thermal insulation properties and can be used as insulation materials for building walls, but their poor flame retardant properties also limit their application to a large extent. Therefore, enhancing the FR of polymers is a significant challenge in ensuring their safety and reliability in a wide range of applications [6,7].

Because of their light weight, low water absorption, high flexibility, good chemical corrosion resistance, high specific modulus and low thermal conductivity, polymers are often used in the field of construction engineering [8,9,10,11,12,13]. In construction engineering, polyvinyl chloride or PP are commonly used as drainage pipes, PP or PE are used as water supply pipe, and polyolefin and polyamide are used as insulation layers in cables. It is well known that polymer is a flammable material. As a result, it is easy to cause a fire and other major risks in the process of using it, which threatens people’s safety. This safety problem has been the focus of much attention [14,15,16,17,18,19].

In recent years, with the gradual development of the field of construction engineering, many researchers have devoted themselves to this field and developed a large number of flame-retardant polymer materials that can be used in construction engineering [20,21,22,23,24,25,26,27,28]. According to the material composition, they can be divided into intrinsic-type and doped-type flame-retardant polymer materials. This paper mainly introduces the mechanism of flame retardancy, the classification of flame-retardant agents, the fabrication of flame-retardant polymer materials and their potential applications in the field of construction engineering.

## 2. Flame-Retardant Mechanism

### 2.1. Burning Mechanism

The polymer combustion process can occur in the condensed phase, gas phase and mesophase. During exposure to external heat flux, the polymer can be pyrolyzed, and the volume of flammable volatiles generated and released into the gas phase increases. These volatile products mix with the oxygen in the atmosphere to form a fuel source, which is ignited and causes the flame to burn. The heat generated in this process is fed back to the condensed phase of the polymer system to maintain the combustion process. In addition, polymer materials tend to melt and flow, forming pools of flammable degradation products, which may constitute a very serious secondary fire hazard as this usually leads to further fire or the burning of surrounding fuel loads [29,30].

The polymer material is heated and automatically forms combustible substances with volatile properties when the temperature rises to a certain value. Combustion will occur when the above substances accumulate to a certain concentration in the air. Specifically, the combustion of polymer materials can be divided into two processes (degradation and combustion in thermal oxygen), including the degradation and decomposition of polymer materials in the condensed phase and the diffusion of their products in the solid and gas phases. At the same time, the degraded products can also be mixed with air, at which point a certain oxidation reaction occurs and a chain reaction begins [31,32].

### 2.2. Flame-Retardant Mechanism

The flame retardancy mechanism is intended to decrease the flammability of the material and slow down the spread of the flame. When the flame is removed, it will quickly self-extinguish, halting the burning process. In the combustion process, the cycle of fuel, heat and oxygen must be broken in order to realize the aim of flame retardancy. In essence, the flame retardant effect is achieved by eliminating or reducing one or more factors, such as fuel, heat and oxygen. It is generally necessary to enhance the thermal stability of polymers and then capture certain free radicals. In order to form a non-combustible protective film, it first absorbs certain heat, then forms a gas isolation layer and finally dilutes oxygen and other combustible gases. The mechanism of flame retardancy is usually physical, chemical or a combination of the two. The first effect is endothermic. Combustion occurs when the temperature reaches the ignition point of a heated combustible material, and flue gas will be generated. In a short period of time, the flame-retardant material can absorb a portion of the heat released by the burning material, causing a strong endothermic reaction. This can somewhat reduce the surface temperature of the burning material, effectively reduce the generation of smoke and prevent the occurrence of the fire spreading. Most flame retardants exert their flame retardant effect via endothermic action. Next is the covering effect. The surface of the combustible material will form a stable foam covering layer when burning. It will isolate the surface of the combustion material from the air and prevent the combustion gas from escaping so as to guard against the fire growing further. Based on the chain reaction theory of combustion, free radicals are necessary to maintain combustion. When the flame retardant reacts with the free radicals produced in the combustion process, the flame density in the combustion zone decreases, and finally the burning rate of the combustible materials decreases until it is completely extinguished. Lastly, a suffocation effect is produced by the non-combustible gas. During combustion, the flame retardant decomposes at high temperatures and releases non-combustible gas. With the increase in volume of non-combustible gas, the concentration of combustible gas is gradually reduced to prevent the continuous combustion process. The condensed phase flame-retardant mechanism refers to the formation of several carbon layers on the surface of the solid phase in order to prevent or delay the generation of combustible gas and the thermal decomposition of the material. The concepts of melt drop flame retardancy and increasing pyrolysis temperature flame retardancy indicate that, under the action of the flame retardant, the fiber material depolymerizes and the melting temperature decreases, which increases the temperature difference between the melting point and ignition point. The fiber material softens, shrinks and melts before pyrolysis and becomes molten liquid drops. Most of the heat is taken away, thus interrupting the process of the heat being fed back to the fiber material. Finally, combustion is interrupted, and the flame extinguishes itself. Alternatively, introducing aromatic or heteroaromatic rings into the macromolecules of the fiber increases the density and cohesion of the macromolecular chains and improves the heat resistance of the polymer used as the fiber; or through macromolecular chain crosslinking cyclization, metal ions form a complex that changes the molecular structure of the fiber, improving the degree of carbonization, inhibiting pyrolysis and reducing the generation of combustible gas [33,34,35,36,37,38].

### 2.3. Standard Tests for Flame Retardancy

#### 2.3.1. Limiting Oxygen Index (LOI)

The LOI value is defined as the minimum oxygen concentration in the flowing oxygen/nitrogen mixture that can keep the flame burning for 3 min or consume 5 cm of sample. According to the ISO 4589 standard, a sample of 80 mm × 10 mm × 4 mm is placed vertically in the center of a glass chimney, and the top of the sample is lit with a burner. LOI testing is considered one of the most important screening and quality control methods in the development of flame-retardant polymers because of the inexpensive process and the relatively small sample size required. However, this test method is not suitable for assessing the true extent of the fire resistance of materials due to the low heat input and high simulated oxygen concentration [39].

#### 2.3.2. Tests for Flammability of Plastic Materials

The UL-94 test set has been used by Underwriters Laboratories to test the flammability of plastics used as equipment and appliance components. The UL-94 vertical test is the most common method used to determine the flammability of plastic materials and their capacity to spread flames. The control burner produces a blue flame, the height of which is 20 mm, at a power of 50 W. The distance between the top of the burner and the bottom of the sample should be kept at 10 mm. Expose the bottom of the sample to the flame for 10 s, then remove it. The time required for the flame to extinguish is denoted as residual flame time, T1. After flameout, the bottom of the sample is exposed to the flame for another 10 s. The time required for the flame to extinguish is denoted as residual flame time, T2, and the time required for the flame’s glow to disappear is denoted as residual glow time, T3. According to IEC 60695-11-10, 5 parallel samples must be tested in each run [39].

#### 2.3.3. Cone Calorimetry

Conical calorimetry is one of the most effective small-scale tests used to measure the combustion behavior of polymer materials. Conical calorimetry tests conform to international standards (ISO 5660). The sample is exposed to a conical radiant electric heater and ignited by an electric spark. The heat release rate (HRR) is calculated by measuring the gas flow rate and oxygen concentration. The peak heat release rate (PHRR) is considered one of the most important parameters when evaluating the fire resistance of materials. The total heat release (THR) value is obtained from the integral of HRR over the time curve. In addition, conical calorimetry can characterize time to ignition (TTI), flameout time (TOF), effective heat of combustion (EHC), mass loss during combustion, production of CO and CO_2_, and total smoke release (TSR). There is no doubt that cone calorimetry provides more detailed fire characteristics and is a powerful tool for simulating material flammability under real-world fire conditions [39].

## 3. Classification of Flame-Retardant Polymer Materials

**Figure 1:**
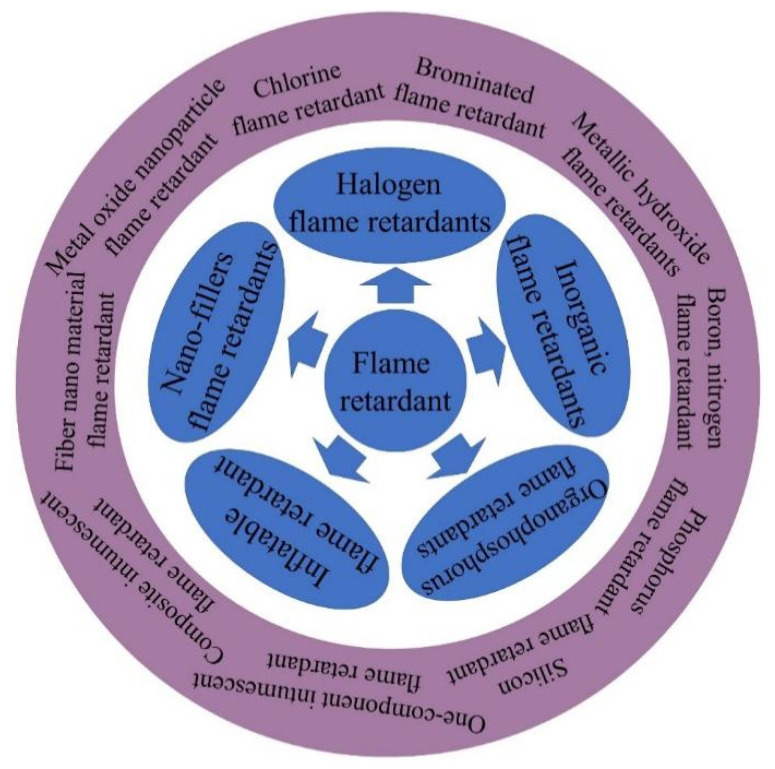
Classification of flame-retardant polymer materials.

**Figure 2:**
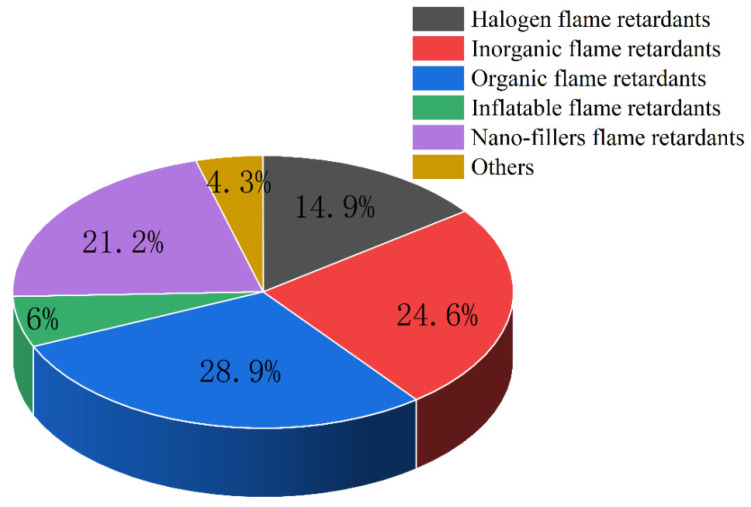
The proportion of representation of each flame-retardant polymer material.

At present, flame retardants can be divided into halogen flame retardants, inorganic flame retardants, organic flame retardants, intumescent flame retardants and nano-filler flame retardants according to the type of fillers. Among these, inorganic flame retardants mainly include metal hydroxides. Inorganic flame retardants include inorganic phosphorus flame retardants, boron flame retardants, nitrogen flame retardants, etc. Organic flame retardants include organophosphorus flame retardants, silicon flame retardants, etc. Figure 1 shows the classification of flame-retardant polymer composites [40,41,42,43]. In the past 30 years, there have been more than 21,000 articles on flame-retardant composites, including more than 3100 on halogen flame retardants, 5100 on inorganic flame retardants, more than 6000 on organic flame retardants, more than 1250 on intumescent flame retardants and more than 4400 on nano-material flame retardants, and the proportions of each are shown in Figure 2.

### 3.1. Halogen-Containing Flame Retardant

Of the flame-retardant polymer materials, halogen flame retardants are flame retardants containing halogen elements. The FR of halogen flame retardants hinges on the variety of the halogen. Fluorinated compounds have greater thermal stability than most polymers, so they do not discharge halogen free radicals in a temperature range similar to or lower than the decomposition temperature of polymer materials. Halogen radicals are readily released and participate in the combustion process because bromine and chlorine demand lower energy to bind with carbon atoms. Halogen flame retardants have the benefits of low cost, high efficiency and easy dispersion. The flame-retardant mechanism is as follows: (1) the flame retardant’s decomposition will absorb heat as the material is heated, thus reducing the temperature of the material; (2) a large amount of hydrogen halide gas is generated, which is denser than air and will gather on the surface of the material to isolate oxygen; (3) a large amount of active free radical HO·is generated in the gas phase combustion region of the material after combustion. Hydrogen halide will then react with it to generate stable inert free radicals, thus realizing the flame retardant effect [44,45,46].

At present, the huge harm caused by halogen flame retardants to the environment is being increasingly questioned and challenged. Halogen-containing flame retardants produce a large amount of toxic smoke and gaseous hydrogen halide during the combustion process. The latter easily accumulates in the food chain and can affect the health of the surrounding environment and human survival. It is thus inimical to the concepts of environmental protection and sustainable development [47,48,49]. Therefore, many researchers are committed to identifying new environmentally friendly non-halogen flame retardants. Some developed countries in Europe have even issued regulations to restrict the use of some brominated flame retardants, which has accelerated the development of other high-efficiency, non-toxic and harmless flame retardants [50].

### 3.2. Inorganic Flame Retardants

Inorganic flame retardants are composed of a high temperature-resistant solution and ultrafine inorganic metal oxides. Inorganic flame retardants mainly add inorganic elements with intrinsic FR to the flame retardant substrate in the form of simple substances or compounds. Then, they are fully mixed with the polymer in the physical dispersion state, and the flame retardant effect is achieved through chemical or physical changes in the gas or condensed phase. Inorganic flame retardants mainly comprise metal hydroxides, inorganic phosphorus series, nitrogen series, silicon series, boron compounds, etc. They have been widely used because of their excellent thermal stability, non-volatility, lasting effect, low price and other characteristics [51].

#### 3.2.1. Metallic Hydroxide Flame Retardants

Aluminium trihydroxide (ATH) and magnesium dihydroxide (MDH) are two typical representatives of metal hydroxide flame retardants, which have the advantages of nontoxicity, good stability and emitting no toxic gas at high temperatures. They can reduce the amount of smoke emitted when plastics combust. They are cheap, widely available and have a good flame retardant effect. Metal hydroxides decompose and release water during combustion, which reduces the temperature of the polymer matrix and reduces the oxygen concentration in the ambient air [52,53,54,55,56]. The metal oxides generated during combustion adhere to the surface of the matrix, forming a protective layer to further inhibit the heat and gas transfer. ATH can significantly reduce the thermal decomposition temperature of PE, PP, polyvinyl chloride and acrylonitrile/butadiene/styrene copolymer (ABS) when added at 40 wt%. It has good flame retardancy and reduces the amount of smoke. Flame-retardant polyolefin with 60 wt% ATH has been widely used in building materials. When an MDH flame retardant is used for PP polymers, it has good flame retardancy when added at 50 wt%. However, the decomposition temperature of MDH is high, at about 340–490 °C, and the heat absorption is low. Therefore, the performance of MDH in inhibiting temperature increase in materials is worse than that of ATH, and the flame retardant effect of polymer carbonization is better than that of ATH. The combination of ATH and MDH can be used to adjust the ratio of the two substances to achieve a strong synergistic effect, which can significantly reduce the application cost of flame retardants. The main problem with common ATH and MDH flame retardants is the large amount of filling required, as this large amount of filling will inevitably lead to the poor fluidity of the materials in resin mixing and molding. This will affect the processing performance and mechanical properties of the materials.

#### 3.2.2. Inorganic Phosphorus Flame Retardants

The inorganic phosphorus system mainly includes red phosphorus, coated red phosphorus and ammonium polyphosphate (APP). Red phosphorus and APP are two kinds of flame retardants with unique properties in the phosphorus series. Red phosphorus has become an important non-halogen flame retardant because of its excellent flame retardancy, low toxicity and synergistic effect with many flame retardants. However, red phosphorus has many disadvantages. Firstly, it easily absorbs water in the air and generates phosphoric acid, phosphite and other substances. Secondly, its compatibility with resin is poor, as well as being difficult to disperse, and it will give rise to segregation settlement. This increases the viscosity of the resin, which makes it difficult to operate and degrades its performance. Furthermore, red phosphorus releases highly toxic PH_3_ gas during long-term contact with air. Dry red phosphorus dust has a high risk of combustion and explosion. In order to overcome the above shortcomings, red phosphorus is coated with organic or inorganic materials and then referred to as coated red phosphorus. The red phosphorus coating can be divided into three types according to the coating materials: inorganic coating, organic coating and organic–inorganic composite coating. For example, red phosphorus can be suspended in an aqueous solution containing an aluminum sulfate coating with ATH. Sodium hydroxide is added to adjust the pH value of the solution to form ATH precipitate on the surface of the red phosphorus, constituting a uniform and dense protective layer. Inorganic coated red phosphorus shows improvements in terms of the ignition point, hygroscopicity and the amount of PH_3_, but its compatibility with resin is still poor. The ignition point is not high enough, and it still produces a certain amount of PH_3_. The organic coating method involves covering red phosphorus with organic matter. At present, thermosetting resin, such as epoxy resin, is used for coating. Organic–inorganic composite coating involves applying a layer of inorganic coating and then a layer of organic coating. The coating of red phosphorus has little effect on the physical and mechanical properties of the products, endows the flame-retardant material with better impact resistance and improves its compatibility with resin. The flame retardant effect of the red phosphorus coating exceeds that of bromine. The coated red phosphorus can also be used in conjunction with many synergistic agents for better results. Polyphosphoric acid is also an important inorganic flame retardant. It has the advantages of a high phosphorus and nitrogen content, good thermal stability, good dispersibility and low toxicity. It is commonly used as the main component in an intumescent flame retardant system. It is widely used in the flame retardation of plastics, rubber, fiber, paper, wood, and so on. Although APP has some of the advantages mentioned above and is widely used, it also has some disadvantages, such as easy moisture absorption in humid air and turning paper yellow and brittle when used for paper processing. It can be microencapsulated to alleviate or overcome the above shortcomings. APP can be microencapsulated by coating it with melamine-formaldehyde resin, Polyplus, epoxy resin, amino resin, etc. [57,58,59,60,61].

#### 3.2.3. Boron-Containing Flame Retardants

Boron compounds belong to another type of inorganic additives with FR [62,63,64,65,66,67,68,69,70]. Of the boron compounds, zinc borate (ZB), such as 2ZnO·3B_2_O_3_·3.5H_2_O, is the most commonly used. The crystal water molecules contained within it and 2ZnO·3B_2_O_3_ are closely combined. When the temperature is above 260 °C, a large amount of external heat is absorbed, and water is released. The released water slows down the endothermic degradation process of the polymer and then turns into steam to absorb a large amount of heat. According to our measurement, every gram of 2ZnO·3B_2_O_3_·3.5H_2_O is decomposed into water vapor and other compounds, and the absorbed external heat is 924 J, which greatly reduces the temperature of the combustor. Meanwhile, the smokeless and nontoxic water vapor produced dilutes the combustible gas and plays a role in isolating the air to a certain extent. At the same time, 2ZnO·3B_2_O_3_·3.5H_2_O will produce a non-volatile B_2_O_3_ glassy material coated on the polymer surface at the combustion temperature. This dense glassy protective layer also plays the role of an isolation film. In most halogenated epoxy resins and some unsaturated polyester (UPR) systems, ZB acts synergistically with antimony oxide, ATH, etc. With the requirement of not reducing the flame retardant grade of the material, it can effectively reduce the material cost and smoke generation. ZB contributes to generating a porous carbon layer during material combustion, and the carbon layer can be stabilized by boron trioxide. At the temperature of polymer combustion, ZB can also generate a hard porous residue similar to glass and ceramic with ATH. It is beneficial for heat insulation and preventing air diffusion into the material. Preventing the formation of droplets can reduce secondary fires caused by said droplets. Therefore, 2ZnO·3B_2_O_3_·3.5H_2_O is a non-toxic and smokeless flame retardant.

#### 3.2.4. Nitrogen-Containing Flame Retardants

In recent years, nitrogen-based flame retardants have attracted widespread attention because of their advantages, such as low toxicity, less smoke generated during fire, high flame retardant efficiency, low corrosiveness, strong thermal stability, high thermal decomposition temperature and environmental protection. Nitrogen flame retardants mainly include APP, guanidine salts, melamine and dicyandiamide compounds, etc. Nitrogen flame retardants produce non-flammable gases in the combustion process, which can block gas phase combustion and thus achieve a flame retardant effect. During the heating process, the -NH_2_ and -NH- in the structure of melamine and its derivatives, guanidine salt and dicyandiamide compounds, usually generate NH_3_ or N_2_, and gradually diffuse into the combustion zone, thus reducing the concentration of oxygen and gaseous fuel. At the same time, the six-membered ring of carbon and nitrogen in the triazine structure can participate in the carbon formation reaction of the material matrix and play the role of increasing carbon formation and strengthening the carbon layer. However, the nitrogen content of nitrogen-based flame retardants is limited. The flame retardant effect is poor, and the compatibility of the material is poor. Therefore, most scholars focus on reducing the thermal conductivity of nitrogen flame retardants and improving material compatibility through modification [71,72,73,74,75].

### 3.3. Organic Flame Retardants

#### 3.3.1. Organophosphorus Flame Retardants

Organophosphorus flame retardants are a kind of flame retardant with good performance. They not only have the double function of flame retardancy and plasticization, but also many varieties, broad uses and a long history. The flame-retardant mechanism of organophosphorus flame retardants is generally as follows: (1) The flame retardant system decomposes to produce volatile radicals (PO, PO_2_, etc.), which can capture the active radicals (H, HO) in the gas phase and stop the chain reaction in the combustion process. At the same time, the non-combustible gas produced by the degradation of organophosphorus flame retardants will dilute the surrounding air and reduce the oxygen concentration. (2) In the condensed phase, the degradation and condensation of organophosphorus flame retardants produces phosphoric acid substances, which can promote the dehydration and carbonization of the matrix to form a carbon layer. The phosphoric acid-containing substances will form a glass-like molten layer on the combustion surface after thermal decomposition, which further inhibits the transfer of heat and gas and achieves the flame retardant effect. Organophosphorus flame retardants and some elements (such as nitrogen, boron, etc.) can play a synergistic flame retardant role, and further improve the flame retardant effect. Recently, researchers have found that the preparation of organophosphorus flame retardants containing sulfur elements in polymer polymerization can quickly enhance the flame retardant effects of polymer materials by means of the synergistic effects of the chemical reaction of phosphorus and sulfur elements in the polymer materials [76,77,78,79].

#### 3.3.2. Silicone Flame Retardants

Silicone flame retardants stand out among the many flame-retardant materials by virtue of their good environmental adaptability and environmental friendliness. Silicone flame-retardant polymer materials have excellent mechanical properties, impact resistance, wear resistance, cold resistance and other characteristics. Combined with silicone flame-retardant materials and other flame-retardant materials, the flame retardant effect is greatly improved. The research on organosilicon flame-retardant materials has mainly improved the flame retardant and smoke suppression effects via enhancing the molecular structure, increasing the relative molecular weight, and blending. Silicon-containing groups have high thermal stability, hydrophobicity and oxidation stability, and excellent flexibility. The silicon-containing groups are introduced into the polymer molecular chain by polymerization, grafting and crosslinking technology. The flame-retardant polymer with silicon possesses the features of FR, heat resistance, oxidation resistance and burn resistance. It also has high moisture resistance and molecular flexibility, and its processability is improved. Recently, silicone flame retardants have mainly included silicone resin flame retardants and polysiloxane flame retardants. The methods include directly introducing a silicone flame retardant into a polymer or embedding a series of polysiloxane segments with functional groups into polymer materials. The organosilicon flame retardant is based on the mechanism of condensed phase flame retardancy, that is, it acts through the formation of a cracked carbon layer and improves the oxidation resistance of this layer. When a silicone flame retardant is added to a polymer material, most of the silicone flame retardant will move to the polymer surface, forming a polymer gradient material with a silicone flame retardant enrichment layer on the surface. Once burned, an inorganic oxygen insulation and insulation protective layer, containing Si and/or Si-C bonds characteristic of polysiloxane, is formed. In this way, the thermal decomposition of polymer materials is inhibited, and the escape of combustion decomposition products is also prevented, realizing the characteristics of flame retardancy, low smoke production and low toxicity [80,81,82,83].

### 3.4. Intumescent Flame Retardant

An intumescent flame retardant is a compound flame retardant that forms an intumescent carbonization layer on the outside of a polymer when heated. It does not contain halogens. It is a new type of environmental protection flame retardant. When the intumescent flame retardant is decomposed by heat, it will produce a dense carbonized layer. This has the functions of heat insulation, oxygen isolation and smoke suppression, thus blocking the combustion process of the polymer, and it has good FR. The intumescent flame retardant consists of three basic elements: acid source, carbon source and gas source. Among the three elements, the acid source is an important element and occupies the largest proportion. The flame retardant elements are contained in the acid source, making it the true flame retardant, and the carbon source and gas source are synergistic agents. The carbon source, also known as the dehydrating agent or carbonization accelerator, represents the basis for forming the carbonized layer of foam. It is mainly composed of polyhydroxyl compounds with high carbon contents, such as pentaerythritol, starch and three azine derivatives. The acid source is generally inorganic acid, or compounds that can form inorganic acid after being heated to a certain temperature, such as phosphoric acid, phosphorus oxychloride, APP, etc. The gas source is also called the foaming source. Commonly used foaming sources include melamine, dicyandiamide, piperazine, ethylenediamine, etc. When an intumescent flame retardant is added to polymer, it should meet the requirements of good thermal stability, compatibility and processing performance. The temperature of thermal decomposition should be higher than the processing temperature of the polymer material. When the polymer burns, a dense carbonization layer is formed on its surface to halt the burning. An expandable flame retardant is a kind of environment-friendly flame retardant that does not produce poisonous, harmful or corrosive gases when decomposed by heat. It has a broad market prospect [56,84,85,86,87,88,89,90,91].

### 3.5. Nano Fillers-Containing Flame Retardants

Prior to the use nano-fillers, microscale fillers were added in the polymer matrix. Although the improvement in thermal stability that they offer is significant, these microcomposites require highly loaded fillers to attain good results. In addition, the obtained highly loaded microcomposites are denser than pure polymer materials, and their mechanical capacities are significantly different. By using nano-fillers instead of microscale fillers, the surface/volume ratio is significantly increased, the interaction between polymer and nano-fillers is enhanced and a smaller filler load is required. The dispersion of nano-fillers in the polymer greatly affects the surface area of the nano-fillers exposed to the polymer, thus affecting the degree of interaction between the nano-fillers and the polymer. The nanocomposites also reportedly show better heat stability, optical and magnetic properties and chemical resistance, and reduced permeability to water, gases and hydrocarbons. In particular, nano-fillers improve FR by enhancing the formation of carbon, reducing the heat release rate, extending the ignition time and reducing the emission of combustible volatiles in fire. Because nano-fillers reduce the mobility of the polymer and the capture of free radicals, nanocomposites exhibit better thermostability than the original polymer, and their FR depends on the geometrical shape and chemical structure of the nano-fillers. Nano-fillers have a large specific surface area/volume ratio, so the interaction between polymer and nano-fillers can be enhanced even with relatively low loading. This makes nano-fillers a better choice because of their low cost and ensures that the original properties of the polymer are not affected by overloading [92,93,94,95,96,97].

## 4. Preparation of Flame-Retardant Polymer Materials

### 4.1. Halogen-Containing Flame-Retardant Composites

The development of halogen-free flame retardants is also undergoing revolutionary changes. Since the beginning of the 21st century, governments and related enterprises have paid great attention to the development of new halogen flame retardants and the modification of existing halogen flame retardants in order to meet the needs of performance, health and environmental protection. This is mainly manifested in the following aspects: (1) The existing halogenated flame retardant product’s structure has been adjusted, and the substitutes of polybrominated diphenyl ethers (PBDEs) have been developed. At present, decabromodiphenylethane and brominated trimethylphenyl indene are used. The combustion and pyrolysis of these two flame retardants does not produce polybrominated dibenzo dibenzofurans. Decabromodiphenylethane has better thermal stability and lower permeability, and can improve the impact strength of the material, which cannot be achieved by other flame retardants [98]. (2) Aiming at resolving the weaknesses of the original halogen flame retardants, a new halogen flame retardant has been developed [99].

Dvir et al. [100] introduced pentabromobenzyl acrylate, and the results show that MDH had no significant effect on the LOI of glass–PP composites, but the burning time decreased significantly with an increase in MDH loading. Halogen-based compounds are another kind of flame retardant widely used in polymer matrix systems to improve the flame retardant properties of polymer composites. Compared with MDH, ATH and other metal hydroxide flame retardants, halogen-based flame retardants have better flame retardant properties. In order to compare the FR of MDH with halogen-based flame retardants, Tai and others [101] introduced a mixture of MDH with phosphate bromide and antimony trioxide to PP composites, respectively, then tested the fire performance of the composites using the LOI test method. It was found that only 30 wt% of the mixture of phosphoric acid bromide and antimony trioxide could achieve the FR of 60 wt% MDH. The halogen flame retardants and their characteristics are shown in Table 1.

The halogen flame retardant is the most widely used plastic flame retardant. These retardants face challenges due to the toxic gas generated by halogen combustion—hydrogen halide gas—and their non-degradability. The further research and development direction of halogenated flame retardants includes adjusting the existing product structure of halogenated flame retardants, and developing substitutes for polybrominated diphenyl ethers, functional halogenated flame retardants and composite halogenated flame retardants.

### 4.2. Inorganic-Containing Flame-Retardant Composites

In recent years, light-weight softwood-based materials have attracted much attention due to their excellent thermal stability and FR. In order to strengthen the applicability of cork in the indoor environment, a new type of light cork material with good thermal stability and FR was prepared via a simple and easy method. Zhai et al. [102] rapidly prepared silica gel composite corks (Cosiae-SP and Cosiae-VP) by immersing corks of different tree species in silicone mucilage via the respiration impregnation method. Silica aerogel was immobilized in the cork cells to form a layered network structure with holes. The thermal stability of Quercus suber corks (COR-S) was better than that of Quercus variabilis corks (COR-V), and cork treated with silica gel had excellent heat stability. The flame retardant and smoke suppression properties of a particleboard made of silica gel composite cork (Cosiae SP and Cosiae VP) were greatly enhanced. The total heat release was reduced by 26.46% and 31.33%, respectively, and the total exhaust volume was reduced by 85.60% and 52.04%, respectively. The physical insulation and adsorption of silica aerogels in cork cells were considered the main causes of the increases in FR and smoke suppression.

Kalali et al. [103] developed a novel wood polymer composite (WPC) flame retardant system using APP and phytic acid-modified layered double hydroxides (Ph-LDH) as raw materials. The addition of Ph-LDH led to the early thermal degradation of WPCs, which produced more thermally stable products and improved the formation of carbon. The combustion performance of WPCs was studied by LOI, UL-94 and cone calorimetry. It was proven that the addition of Ph-LDH had synergistic effects on the combustion performance of WPCs. Therefore, WPC has good FR and mechanical properties and can be used in buildings or furniture requiring high FR.

**Figure 3:**
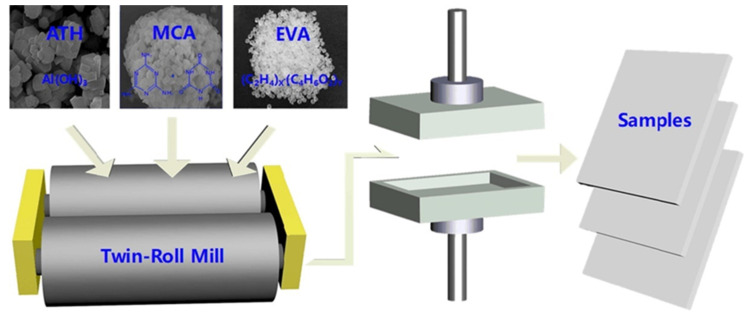
Schematic illustration of synthetic route of the flame retardant EVA composites [104].

Ethylene vinyl acetate (EVA) is a widely used material, but its high flammability seriously hinders its application as a product material. Xu et al. [104] prepared high-flame-retardant EVA composites via the melt blending and template method (as shown in Figure 3). The combustion results show that EVA-60-40, with 60 wt% ATH and 40 wt% MCA, formed the best thermal insulation layer, which was beneficial to FR. In addition, EVA-60-40′s highest LOI value of 27.5% and its UL-94 V-0 grade were compared with other components, indicating its non-flammability. These results show that the addition of ATH and MCA had a synergistic effect, and the prepared EVA-ATH-MCA composite has broad applicability in the construction field.

Cinausero et al. [105] studied the synergistic effect of nano-oxide and ammonium polyphosphate (APP) with polymers such as polystyrene (PS) and polymethylmethacrylate (PMMA). The results of cone calorimetry show that the synergistic application of silicon and an APP flame retardant can reduce the peak heat release rate, reduce the opaque smoke produced and improve the LOI value. Nazare et al. [106] studied the synergistic effects of the application of APP, melamine phosphate (MP) and an ATH silicate layer of PET resin. They found that MP underwent the greatest peak heat release rate reduction (PHRR), followed by APP and ATH.

**Figure 4:**
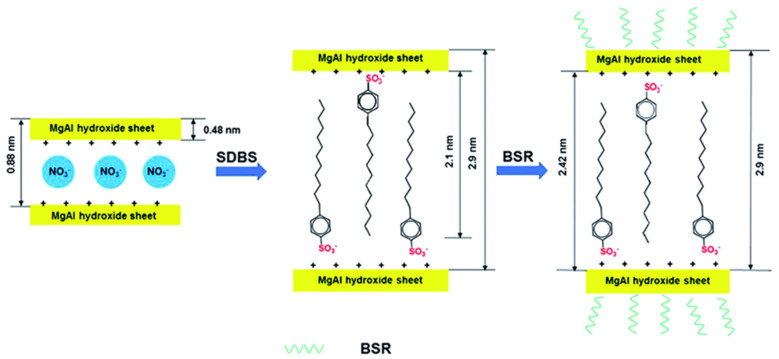
Schematic illustration of the preparation process of DBS-LDH/BSR [107].

Jiang and others [107] developed a new synergistic flame retardant system, composed of layered double hydroxide (LDH) modified by boron-doped silicone resin (BSR) and sodium dodecylbenzene sulfonate (SDBS), denoted as DBS-LDH/BSR (as shown in Figure 4). The synergistic application of a flame retardant to a polycarbonate can promote the formation of polycarbonate carbon, improve the flame retardant grade as assessed via a vertical combustion test, increase LOI and reduce the peak heat release rate in the cone calorimetry test. The synergistic FR was attributed to the formation of -Si-O-, -Si-C- and -Si-O-B- bonds, and other thermal insulation layers or physical barriers. Tawiah’s group [108] carried out similar research. The use of synergistic flame retardants in polylactic acid reduced the degradation rate of polylactic acid and increased the formation of carbon in a thermal gravimetric analyzer (TGA). At the same time, the vertical combustion test rating increased, the LOI rating increased and the peak heat release rate under cone calorimetry decreased. The inorganic flame retardants and their characteristics are shown in Table 2.

### 4.3. Organic-Containing Flame-Retardant Composites

**Figure 5:**
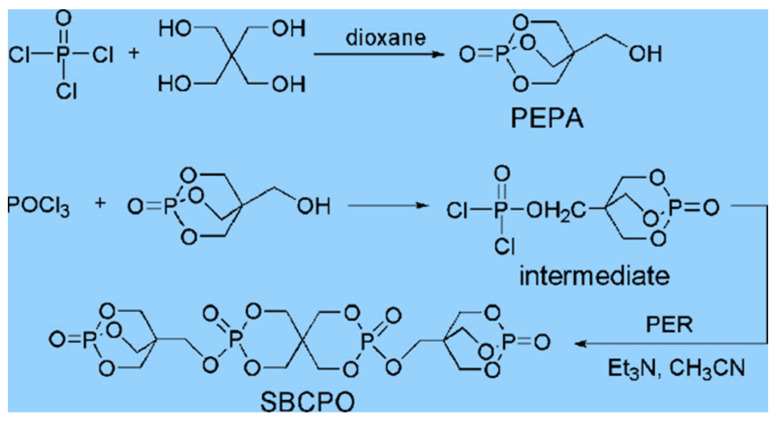
Synthesis route of SBCPO.

Among all organic flame retardants, phosphorus series flame retardants have the best effects. Tian et al. [101] prepared a new phosphate ester flame retardant SBCPO (as shown in Figure 5) using 2,6,7-Trioxa-1-phosphabicyclo [2,2,2] octane-4-methanol (PEPA). It was found that when the mass fraction of flame retardant reached 25.0 wt%, the corresponding LOI was 30.5%. In addition to phosphorus organic flame retardants, organic flame retardants containing multiple flame retardants can also optimize the FR of polymer materials, and the synergistic FR between multiple elements is improved. This can increase the LOI of polymer materials to a greater extent.

**Figure 6:**
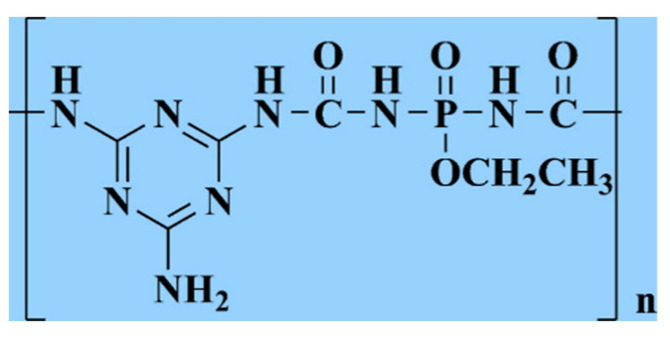
Structure of PMPC.

LV and others [109] used phosphorus oxychloride, ethyl carbamate, ethanol and melamine to prepare organic flame retardants (as shown in Figure 6) containing phosphorus and nitrogen at the same time, and then added them to epoxy resin. It was found that when the mass fraction of the organic flame retardant was 20 wt%, the LOI of the epoxy resin was 28.0%. In addition to using a single element to modify the material, the synergistic effects of multiple elements can also be used to modify the flame retardancy of the material.

Meng-E’s group [110] fabricated a novel kind of inorganic silicon flame retardant using nano-silica and APP as raw materials. The experimental results show that nano-silica and APP had synergistic effects on flame retardancy performance. It was demonstrated that the LOI increased from 19.6% to 23.3%, and the thermal conductivity decreased, when the mass fractions of silica and APP were 6 wt% and 8 wt%, respectively. This greatly enhanced the FR of the material.

**Figure 7:**
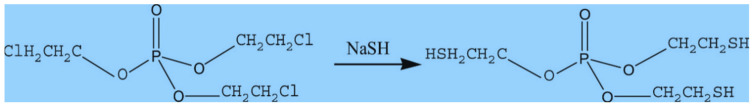
Synthesis scheme of TMEP.

Luo et al. [111] prepared a new phosphorus and sulfur material containing curing agent and flame retardant tris (2-mercaptoethyl) phosphate (TMEP, Figure 7), derived from tris (2-chloroethyl) phosphate (TCEP) and sodium bisulfate. The synthetic route is shown in the figure. TMEP was mixed with triethylenetetramine and epoxy prepolymer at a certain ratio and cured to obtain the sample. The FR of the cured epoxy resin was determined by UL-94 vertical combustion and the LOI test. The results show that the cured epoxy resin with 21.6 wt% TMEP can reach UL-94 V-0 grade, and its LOI value was 29.2%.

**Figure 8:**
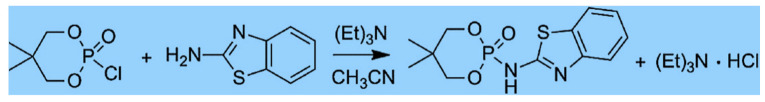
Synthetic route of DOP-ABZ.

Jian et al. [112] synthesized benzothiazole flame retardant DOP-ABZ using 5,5-dimethyl-2-chloro-1,3,2-dioxophosphorus heterohexanoyl phosphate (DOP) and 2-aminobenzothiazole (ABZ) as raw materials (synthetic route of DOP-ABZ is shown in Figure 8). At the same time, a flame retardant epoxy resin (IFR-EP) with different DOP-ABZ contents was prepared. Compared with the common epoxy resin, the thermal stability of IFR-EP was reduced. Epoxy resin with 20 wt% DOP-ABZ (EP/20 wt% DOP-ABZ) was rated UL-94 V-0, with a high LOI of 28.3%. At the same time, cone calorimetry testing showed that the peak heat release rate (PHR) decreased from 1139.7 kW/m^2^ to 238.9 kW/m^2^ compared with EP, and the production of flue gas and toxic gases (including CO and CO_2_) was also significantly reduced. In addition, the mechanical properties of EP/20 wt% DOP-ABZ were also improved to a certain extent. Finally, the flame-retardant mechanism of DOP-ABZ was studied. DOP-ABZ produced phosphoric acid, which dehydrated and carbonized the epoxy molecular chain, thus promoting carbonization. At the same time, the nitrogen/sulfur intermediate released non-flammable gas, which made the formed carbon layer expand, thus achieving a flame retardant effect.

**Figure 9:**
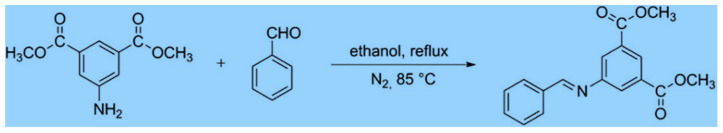
Synthesis route of 5-(benzylidene-amino)-isophthalic acid dimethyl ester (BA).

Wu et al. [113] synthesized the Schiff base product 5-(benzyl-amino)-dimethyl phthalate (BA) using 5-amino-dimethyl phthalate and benzaldehyde as raw materials (as shown in Figure 9) and used this to improve the flame retardant properties of polyethylene terephthalate (PET). The tests show that the BA unit can be self-crosslinked during combustion, which improves the melt viscosity of PET and can promote the formation of the carbon layer, giving PET excellent FR and anti-drop properties. When the BA content was only 7.7 wt%, the LOI value of the flame retardant PET increased from 22.0% to 31.0%, reaching UL-94 V-0 grade. The results show that the aromatic Schiff groups can form a nitrogen-containing crosslinking network (triazine structure) at high temperatures, which can be further transformed into a dense carbon layer. The carbon layer acts as an effective barrier, blocking oxygen, preventing the evaporation of combustible gases and isolating unburned polymers.

**Figure 10:**
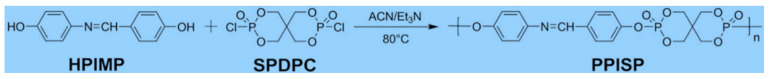
Synthetic route for PPISP.

Zhao et al. [114] designed and synthesized a new type of organophosphorus polymer flame retardant, poly (hydroxyphenylaminomethylphenol)pentaerythritol diphosphate (PPISP), which contains both the Schiff base and the structure of spiropentaerythritol diphosphate. The synthesis route of PPISP is shown in Figure 10. When PPISP was added to the unsaturated polyester (UP) matrix, the initial decomposition temperature (Td) and glass transition temperature (Tg) of the cured UP thermosetting resin containing 20 wt% PPISP were much higher than those of the pure UP. At the same time, the LOI value of the thermosetting composite was 28.2%, reaching UL-94 V-0 grade, and the PHRR value was reduced by 60% compared with that of pure UP. These results indicate that PPISP significantly improved the FR of UP. In addition, the flame retardant UP (FRUP) also demonstrated excellent sustained flame retardancy and water resistance. These results indicate that the Schiff base and spiral bisphosphate structures jointly promoted the thermal stability of UP and acted as a protective layer during pyrolysis and combustion.

Xu et al. [115] synthesized a kind of flame-retardant curing agent MP, with vanillin, 4,4′-diaminodiphenylmethane (DDM) and 9,10-dihydro-9-oxa-10-phosphenanthrene- 10-oxide (DOPO) as raw materials, which was mixed with APP to enhance the FR of bisphenol A. When the contents of MP and AAP in the epoxy cure were 2.2 wt% and 5.1 wt%, respectively, the LOI value of the epoxy cure increased from 24.5% to 29.4%, and it passed the UL-94 V-0 grade. The phosphorus-containing structure of MP was conducive to the formation of a high-thermal-stability phosphorus-rich carbon layer, which effectively blocked the transfer of heat and oxygen. At the same time, APP released non-flammable nitrogen gas when heated, diluting oxygen and other flammable gases. Therefore, the synergistic flame retardant effects of MP and APP gave the cured product excellent flame retardant properties.

Gouri et al. [116] synthesized hexaglycidyl cyclotriphosphazene (HGCP) by the solution mixing method. Flame-retardant EP composites were prepared by blending the compound with diglycidyl ether of bisphenol A (DGEBA). The results showed that the 20 wt% HGCP EP composites had good flame retardant properties, reaching V-0 grade. In addition, HGCP can work in the condensed phase and gas phase, so it can significantly inhibit the generation of smoke. Compared with the thermosetting materials blended with 4,4-methylenediphenylamine (MDA), HGCP had a greater high-temperature thermal stability and charring rate, which improved the FR of the composites. The organic flame retardants and their characteristics are shown in Table 3.

### 4.4. Expansion Flame-Retardant Composites

Nowadays, expandable flame retardant systems have been widely explored, but there are still many problems to be solved, such as moisture absorption, the ease of precipitation, the unstable carbon formation and other shortcomings. Many researchers have put forward a series of solutions to this problem, such as the improvement and development of the acid source, the nitrogen source and the carbon source, and the coordination of flame retardant technology with other flame retardants.

Liu et al. [117] synthesized a new intumescent flame retardant system that was composed of a carbonizing agent (CA), APP and organic modified montmorillonite (OMMT). When 20 wt% of the new flame retardant was added, the LOI of the polymer reached 31.3%, and the vertical combustion test achieved UL-94 V-0 grade.

Lewin et al. [53] added manganese acetate, manganese sulfate, zinc acetate and other metal ion compounds to the intumescent flame retardant PP and conducted LOI and vertical combustion tests to study the synergistic flame retardant effect. The results show that the co-effect of the metal ions on the flame-retardant materials was different with different valence states. The effect of divalent ions was mainly to catalyze the dehydration and crosslinking reaction, while that of polyvalent ions was mainly to catalyze oxidation.

Wang et al. [118] designed an efficient coating method for preparing flame-retardant thermoplastic RR (TPO) sheets. This method can effectively overcome the negative effects of the high loading of a flame retardant on the mechanical properties and processability of materials. A coating (thickness: 120 μm) with expanded flame retardant and TPO (film-forming resin) can help the TPO sheet (the content of expanded flame retardant in the sheet is only 13.06%) to reach V-0 grade in the UL-94 test. The LOI was 30.5%, and the heat release rate measured by cone calorimetry was greatly reduced. In comparison with the traditional bulk flame retardant method, the TPO flame-retardant sheet fabricated via the above method achieved the same flame retardant performance (loading with 36 wt% flame retardant in the traditional bulk addition method resulted in a UL-94 V-0 level). Due to the lower loading and higher flexibility, this has a broad application prospect in the construction field. The expandable flame retardants and their characteristics are shown in Table 4.

### 4.5. Nano Fillers-Containing Flame-Retardant Composites

Laoutid et al. [34] summarized the flame retardant properties of polymer composites obtained by adding nano-fillers to a polymer matrix and accounted for the flame-retardant mechanisms of various nano-fillers. The results show that the thermal and volatile barriers formed by the nano-clay particles migrated to the surface of the polymer, which improved the flame retardant properties of the nano-clay/polymer composites. Fibrous nanomaterials, such as carbon nanotubes, multi-walled nanotubes and siloxanes, can enhance the FR of the composites by enhancing the formation of carbon. Nano-clay, carbon nanotubes and siloxane can improve the FR of the composites, but the effect is not obvious, while nano-metal oxides and hydroxides are effective.

Zhang et al. [119] proved that the optimal loading of nano-clays in ethylene–propylene–diene monomer rubber (EPDM) with an aluminum hydroxide composite was 3 wt%, which maintained a sufficient LOI value and a V-0 grade in the UL-94 test. Laachachi’s group [120] introduced titanium oxide and iron oxide nano-fillers to PMMA. The combustibility of the two kinds of materials was assessed by cone calorimetry. They found that 20 wt% titanium oxide nano-fillers reduced the peak heat release rate by 50%, while the presence of the same amount of ferrous oxide nano-fillers reduced the peak heat release rate by only 35%. The addition of titanium oxide nano-fillers to PMMA significantly increased the ignition time, while the addition of ferrous oxide nano-fillers had no effect on the ignition time of PMMA.

Mishra’s group [121] observed that adding nano-MDH to PP composites significantly improved their FR, which was tested by evaluating the combustion rate per second of nano-MDH-PP composites. The results show that, compared with pure PP, the FR increased by 35% when 12 wt% of nano-MDH was dispersed. Murariu’s group [122] fabricated polylactic acid (PLA)-based nanocomposites by adding nano-calcium sulfate into the PLA matrix. Two organic modified layers of silicate (OMLS) were added into the nano-sized calcium sulfate/polylactic acid composites to further improve the flame retardant performance of the composites. The combustion properties of the OMLS nano-sized calcium sulfate/polylactic acid composites were studied by cone calorimetry and UL-94 tests. Adding OMLS and nano-calcium sulfate into the PLA matrix can improve the combustion performance of the nano-calcium sulfate/PLA composite.

Hossein [123] used reduced graphene oxide (RGO), melamine and microencapsulated ammonium polyphosphate as synergists, and an intumescent flame retarded polyurethane (IFRPU) composite was prepared. When 18 wt% IFR and 2 wt% RGO were added, the limit oxygen index increased from 22.0 to 34.0. In addition, the addition of RGO to the IFRPU composite manifested excellent droplet resistance and helped achieve a UL-94 V0 grade. GO was modified with 4,4-diaminodiphenylmethane (DDM) and then introduced in situ into phosphamide oligomers to prepare nanocomposite flame retardants (FRS-RGO) containing stripped graphene. Subsequently, flame retardants (FRS-FGO) and compatible graft maleic anhydride were added to polypropylene (PP). The addition of FRS-FGO also improved the crystallization and fire resistance of PP composites, including increasing crystallization temperature (11.4 °C, decreasing peak heat release rate (by 66.9%) and total heat release rate (by 24.4%), and decreasing fire growth rate index (by 73.0%) [124]. The flame-retardant composites containing nano-filler and their characteristics are shown in Table 5.

## 5. Polymer-Based Flame-Retardant Composites and Application in Construction Engineering

The matrix of polymer composites can be roughly divided into the thermoplastic matrix and the thermosetting matrix. Thermoplastic and thermosetting matrixes burn quickly, and the FR of these polymers can be enhanced via three possible methods: (1) through introducing flame-retardant fillers into the polymer matrix; (2) through the reacting, or chemical incorporation of, flame retardants with, or into, the main chain of the polymer; (3) through expanding the system. It is reported that nano-fillers can enhance the FR of polymer materials. Therefore, adding nano-fillers is another way to enhance the FR of polymers.

### 5.1. Thermoplastic Flame-Retardant Composites

Most thermoplastic polymers are combustible and drip water during combustion to help limit the flame. The fire performance of these thermoplastic composites has been improved in various ways [125,126]. Suppakarn et al. [127] researched the effects of adding MDH and ZB as flame retardants in sisal–polypropylene (S-PP) composites. The flammability of these composites was examined via horizontal combustion testing. The results show that the combustion rate of S-PP composites was higher than that of pure PP. The combustion rate decreased with the increase in MDH concentration when MDH was added into the PP matrix.

The ZB has no effect on the combustion rate of PP, but with the increase in ZB concentration, the carbon generation rate increases. However, when ZB and MDH were introduced into a PP matrix together, there was no synergistic effect. Boric acid and MDH or ZB were introduced into sawdust/rice husk–polyethylene composites, and similar results were obtained. The rice husk-filled composite burned more easily than the wood chip-filled composite. When 25% rice husk was used instead of MDH, a 50% increase in FR was achieved, and the same tendency was also discovered for the wood chip-filled composite. The combustion rate of the combination of MDH and boric acid was lower than that of the combination of MDH and ZB, while the LOI value of the combination of MDH and ZB was higher than that of the combination of MDH and ZB [128].

### 5.2. Thermosetting Flame-Retardant Composites

Manfredi’s group [129] fabricated some composites with mod-acrylic acid and UPR as substrates, and jute, flax, sisal and glass as reinforcements, and compared the FR of these composites. The results show that the heat release of the flax fiber-reinforced composites was the highest, while the heat release of the glass fiber-reinforced composites was the lowest. At the same time, the flax composite material had a larger flash zone, which indicated that its escape or fire extinguishing time was the longest, and the jute composite material released the least volatile smoke, and its burning speed was faster than other natural fiber composite materials. During combustion, mod-acrylic acid formed a focal layer not found in UPR resins. The fire risks of each composite material were compared by drawing the curve of total heat with peak heat release rate and ignition time. It was found that these glass fiber-reinforced UPR composites, which are not flame retardant, have the lowest fire risk compared with other composites [130].

Similar to thermoplastic composites, the flame retardant performance of thermosetting composites can be improved in different ways. The borax–boric acid mixture has a certain flame retardant effect. In addition to the usual catalytic action of carbon formation, it possesses a low melting point, and produces a glassy film surrounding the combusting parts when exposed to fire. Baysal’s group [131] prepared vinyl monomer–wood composites by treating sapwood with a mixture of 1 wt% borax and boric acid (1:1). The vinyl monomer–wood composites were prepared by using styrene, methyl methacrylate and a mixture of styrene and methyl methacrylate (50:50). The FR of the composite was evaluated using the combustion weight loss method. The results indicate that the mass loss rate of the composite decreased significantly when the sapwood was treated with a mixture of 1 wt% borax and boric acid (1:1). Fernandes, V.J.’s group [132] introduced decabromodiphenyl combined with antimony trioxide as an additive to UPR to improve the FR of sisal–polyester (SSP) composites. The additive was mixed with the polymer matrix in a high shear manner, and then the curing catalyst was added. The FR values of SSP and SSP combined with a flame-retardant material were tested by UL-94 test. The results showed that the SSP composite burns completely, and the flame retardant SSP composite can self-extinguish in 0.72 s, which makes the safety standard of the polymer system very high (V-0 level).

### 5.3. Application of Flame-Retardant Polymer Materials in Construction Engineering

In social development, the status of the construction industry is very high, and the FR of the materials used in the construction industry is the guarantee of the safety of residents. One of the most important goals of the construction industry is to prepare polymer materials with excellent flame retardant properties, and apply them as building engineering materials, interior decoration materials, etc., for the sake of safety and cost reduction. The polymer materials used in the field of construction engineering include general plastics, engineering plastics, thermosetting plastics, etc. Among them, general plastics include PE, PP, polystyrene, PVC, etc.; engineering plastics include phenolic resin, urea formaldehyde resin, polyurethane, etc.; and thermosetting plastics include epoxy resin, UPR, etc. These materials are widely used in the manufacturing of walls, ceilings, floors, cables and other products, but their flame retardancy and fireproof performance are poor, which means they struggle to meet the practical requirements. Therefore, it is necessary to improve the properties of flame-retardant polymer materials by imbuing them with excellent comprehensive properties. Qualified building materials should have the following characteristics: (1) First of all, they should be non-combustible or have good flame retardant properties. Building materials must have high safety qualities, good fire performance ability and a long service life. (2) Second, the heat insulation effect must meet the national strategic requirements of energy conservation and environmental protection. Building materials should have low thermal conductivity, a large heat storage coefficient and high bond strength. (3) Moreover, a short construction period and good water and crack resistance can effectively reduce the project’s cost, improve the enterprise benefit, ensure product quality and fulfill the national product acceptance quality standard.

Mitchell et al. [133] compared extruded polystyrene foam with rice husk/mycelium biological plate and found that the biomass system is expected to have better flame retardancy due to the presence of carbonaceous coke and embedded silica in the combustion process [133,134]. The results show that the values of PHRR and total smoke production (TSP) were significantly reduced by 73.5% and 96.6%, respectively. In addition, Saad’s group also studied some parameters of rice husk smoldering behavior, including initial temperature, particle size and gas products. By investigating the free diffusion of the rice fuel bed during convective heating, they observed the transformation from smoldering to open flame combustion [135]. Ying et al. [136] also studied the preparation of straw magnesium cement (SMC) from rice straw, another bio-based isolation material. In addition, studies have proven that rice straw is easier to self-heat than rice husk, which triggers the occurrence of spontaneous combustion in biofuels [137]. This difference is caused by chemical composition, physical properties, bulk density and surrounding environment.

Flame-retardant poly materials are very important in the construction industry. These flame-retardant polymer composite materials have huge application prospects in the field of construction engineering and can be used in walls, floors, cables, suspended ceilings, and so on.

## 6. Summary and Outlook

This review summarizes the combustion and flame-retardant mechanisms of polymers, classifies flame retardants in detail and introduces the preparation methods of some new flame retardants. At present, these flame-retardant materials can be used alone or as synergists and have good flame retardant properties and great potential in the field of building engineering. However, most of them are still in the stage of laboratory research. In order to ensure their continuous automatic production and commercialization, significant improvements in cost, process and method are needed. Good synergistic effects can be achieved among halogen, inorganic, organic, intumescent and nano-particle flame retardants, because the interface interactions and multi-functional synergistic effects between these elements are different. In order to improve the interface compatibility between matrix and filler, other new methods can be further explored in the context of the surface modification of filler/matrix. Therefore, further explorations of the synergistic effect of and interfacial compatibility between flame retardants are an important means to further improve the FR of materials in the future. In future research, we should try our best to explore the process from laboratory to industrial application and strengthen the application of these flame-retardant materials in the field of construction.

## Figures and Tables

**Table 1:** Halogen flame retardants and their characteristics.

Composites	Characteristics	References
Decabromodiphenylethane and brominated trimethylphenyl	Thermal stability, low permeability and excellent impact resistance	[98]
Pentabromobenzyl acrylate	Better flame retardant performance	[100]
Phosphate bromide/antimony trioxide/PP	Low filler content and high flame retardancy	[101]

**Table 2:** Inorganic flame retardants and their characteristics.

Composites	Characteristics	References
Cosiae-SP and Cosiae-VP	Excellent thermal stability and smoke suppression ability	[102]
WPCs	Better flame retardant performance, no pollution and environmental protection	[103]
EVA-ATH-MCA composite	Wide range of applications	[104]
APP/PS and APP/PMMA MP/ATH	Low PHRR and smoke suppression ability	[105,106]
DBS-LDH/BSR	No pollution and low PHRR	[107]

**Table 3:** Organic flame retardants and their characteristics.

Composites	Characteristics	References
SBCPO	Low content and high flame retardancy	[101]
PMPC	Blending and synergistic effect	[109]
APP/SiO_2_	Processing convenience and excellent flame retardant properties	[110]
TMEP	Excellent flame retardant properties	[111]
DOP-ABZ	Good thermal stability and less smoke	[112]
BA/PET	Better flame retardant properties	[113]
PPLSP	Good durability and excellent water resistance	[114]
MP/APP/EP	Synergistic flame retardant properties	[115]
HGCP/EP	Excellent thermal stability and smoke suppression ability	[116]

**Table 4:** Expandable flame retardants and their characteristics.

Composites	Characteristics	References
CA/APP/OMMT	Low content and high flame retardancy	[117]
Intumescent flame retardant PP	Metal ions	[53]
TPO	Processing convenience and low PHRR	[118]

**Table 5:** Flame-retardant composites containing nano-filler and their characteristics.

Composites	Characteristics	References
Nano-clay/polymer composites	Improve the flame retardant properties of composites	[34]
Nano-clays/EPDM/ATH	Processing convenience	[119]
Titanium oxide and iron oxide nano-fillers	Processing convenience and low PHRR	[120]
Nano-MDH/PP	Improved flame retardancy	[121]
PLA/CaSO_4_/OMLS	Improved flame retardancy	[122]
IFRPU	Excellent droplet resistance and low PHRR	

## Data Availability

No copyright is required since it is an open source journal.
